# Fingolimod‐induced bladder lymphoma in a patient with multiple sclerosis

**DOI:** 10.1002/ccr3.7280

**Published:** 2023-04-24

**Authors:** Saeed Vaheb, Elham Moases Ghaffary, Vahid Shaygannejad, Omid Mirmosayyeb

**Affiliations:** ^1^ Isfahan Neurosciences Research Center Isfahan University of Medical Sciences Isfahan Iran; ^2^ Department of Neurology, School of Medicine Isfahan University of Medical Sciences Isfahan Iran

**Keywords:** bladder lymphoma, Fingolimod, multiple sclerosis

## Abstract

**Key Clinical Message:**

Malignancies were reported in some studies following taking Fingolimod. We reported a case of bladder lymphoma after taking Fingolimod. Physicians should consider the carcinogenic effects of Fingolimod in long‐term use and replace it with safer medicines.

**Abstract:**

Fingolimod is a medication with a potential cure to control multiple sclerosis (MS) relapses. Here we describe a 32‐year‐old woman with relapsing–remitting multiple sclerosis who developed bladder lymphoma induced by long‐term use of Fingolimod. Physicians should consider the carcinogenic effects of Fingolimod in long‐term use and replace it with safer medicines.

## 1 INTRODUCTION

Fingolimod (or Gilenya) is a type 1 sphingosine‐1‐phosphate receptor antagonist that reduces the sensitivity of auto‐aggressive lymphocytes to cellular signals to exit from secondary lymphoid tissues and thus reduces the recirculation of these lymphocytes in the circulatory system, and probably prevents multiple sclerosis (MS) relapses and other clinical disturbances. Moreover, prior studies have shown that Fingolimod, as a lipophilic molecule that has the ability to cross the blood–brain barrier, can possess neuroprotective and restorative effects by interfering with the mechanism of sphingosine‐1‐phosphate receptors.^1^, ^2^

As of 2010, the United States Food and Drug Administration (USFDA) has approved Fingolimod for the treatment of relapsing multiple sclerosis (RMS) patients over the age of 10 years. Furthermore, it was the first drug approved for use in pediatrics in the United States owing to its efficacy and appropriate safety.^3^, ^4^

Despite these factors, there has been evidence in previous publications suggesting that long‐term use of Fingolimod can rarely increase cancer risk or trigger it in patients with multiple sclerosis (pwMS). In a study conducted by Peter Alping et al.,^5^ analyses of 1620 individuals who consumed Fingolimod in a cohort were performed in order to assess the risk of cancer. A total of 28 cases of cancer were diagnosed in the study. In the entire population of Fingolimod users, the following malignancies were reported: breast cancer in women, prostate cancer in men, melanoma, lymphoma, non‐melanoma skin cancer, and cervical intraepithelial neoplasia.

Even so, few studies have investigated cancer in patients receiving Fingolimod^6^, ^7^; this may be due to the rarity of this adverse effect in the patient population. As of yet, there has been no reported case of bladder lymphoma in pwMS taking long‐term Fingolimod. The purpose of this study is to present the diagnosis and treatment process of the first MS patient treated with Fingolimod with bladder lymphoma.

## 2 CASE HISTORY

The case was a 32‐year‐old woman diagnosed with relapsing–remitting multiple sclerosis (RRMS) in 2010, with primary symptoms of sensory disturbances and an initial expanded disability status scale (EDSS) of 1.5. The disease‐modifying therapy (DMT) used by the patient was interferon β‐1a 30 mg (Brand name CinnoVex) until 2014 when it was changed to Fingolimod following reduction of efficacy, and the patient had been taking Fingolimod regularly for 7 years. The drug was taken five times a week by taking a single capsule of Fingolimod 0.5 mg. MS was stabilized by taking Fingolimod, and the patient's EDSS decreased to 0. The patient did not experience any relapses while on Fingolimod, and the medication was still being taken until 2021. It was found that the patient had no history of primary and secondary smoking and that she had not been exposed to carcinogenic agents in her occupation or environment. There was no history of cancer, tumor, and malignancy in her family.

**FIGURE 1:**
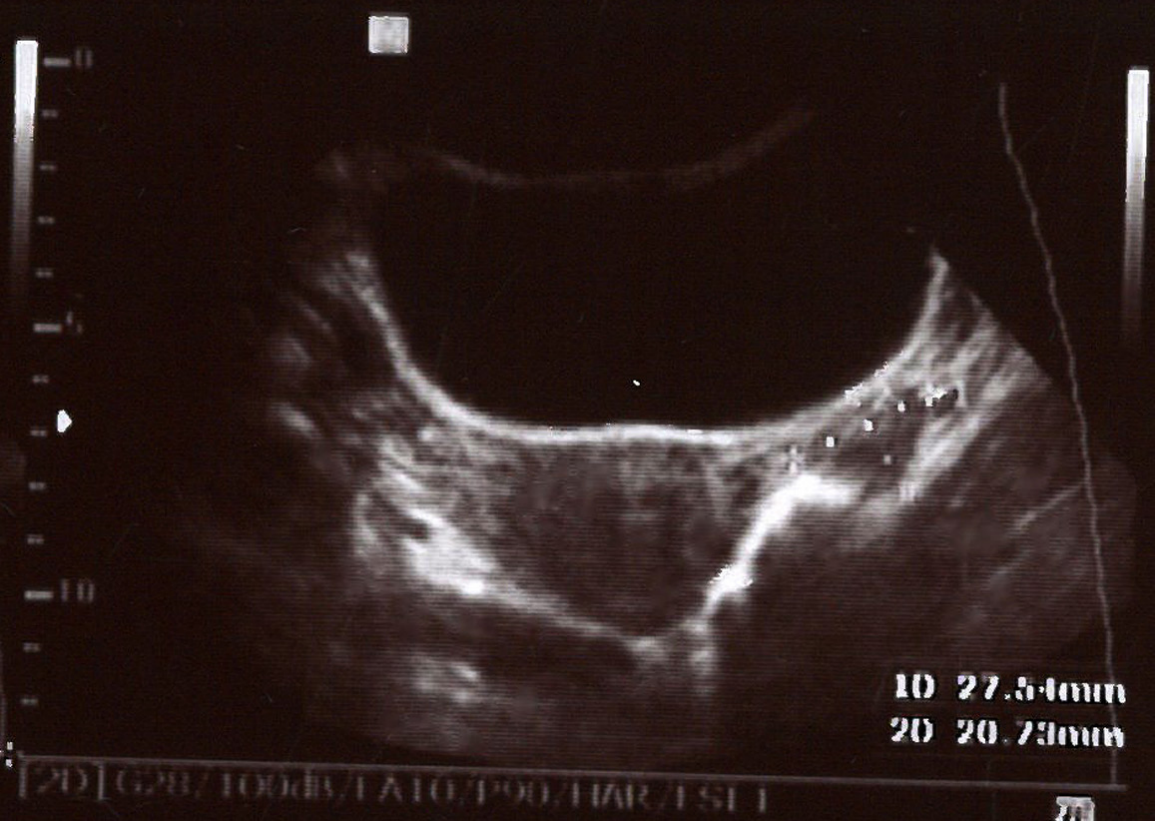
In the sonography, a 16 × 32 polypoid lesion is seen in the posterior wall of the bladder (in the center of the Figure).

**FIGURE 2:**
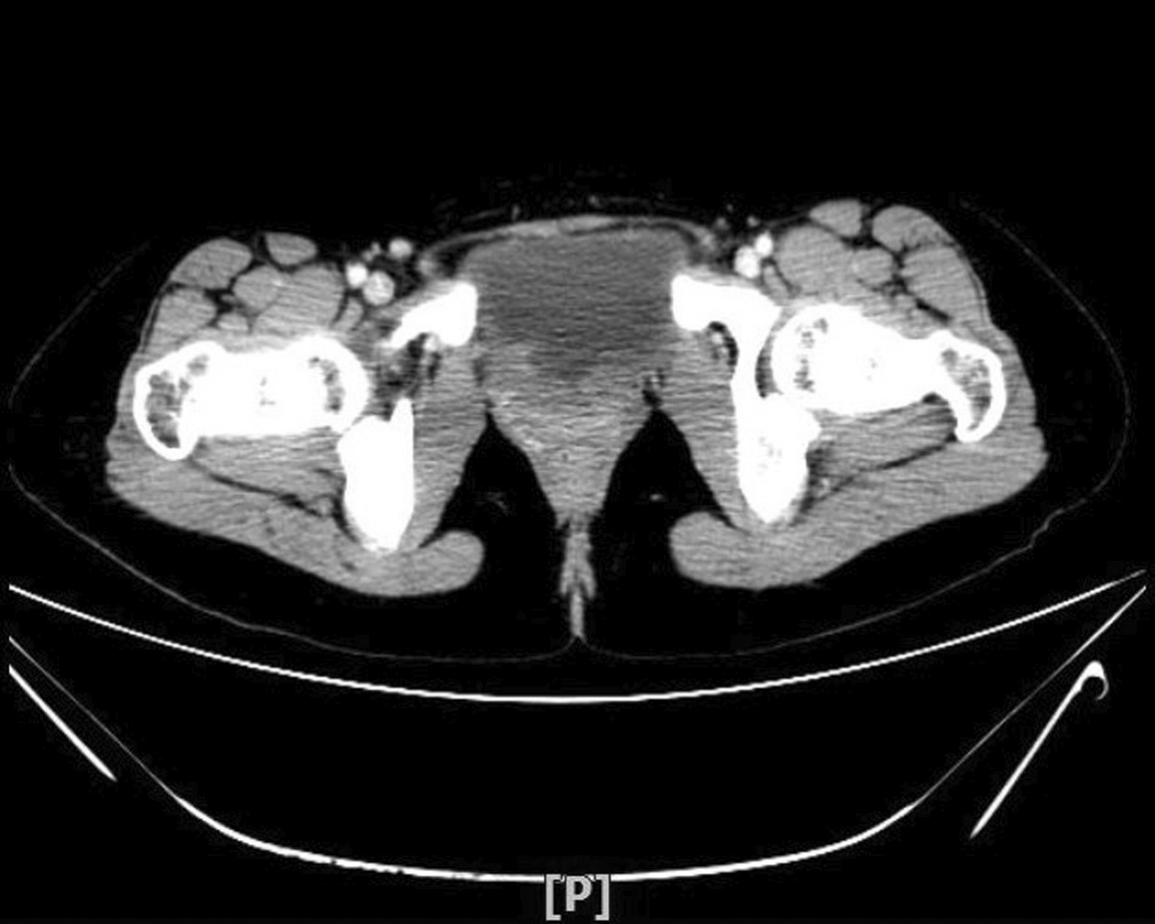
In the CT scan, a small polypoid lesion in the right side of the bladder base (5 mm) with no perivesical infiltration.

**FIGURE 3:**
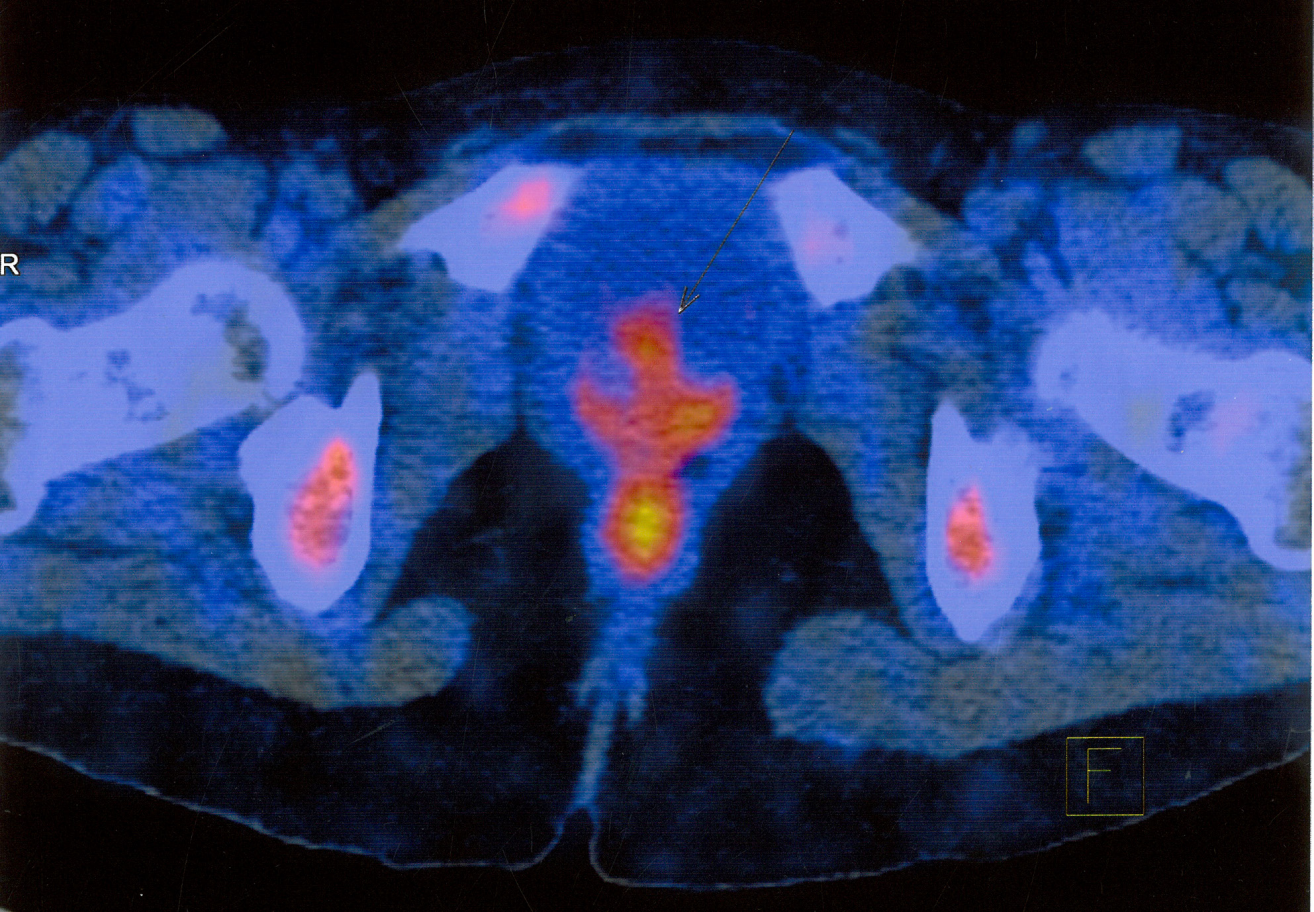
In the PET scan of the pelvis, red flecks indicate the location of the lymphomatous lesion.

The patient presented with symptoms of hematuria, blood clots in the urine, and night sweats, in 2020. An abdominal and pelvic sonogram was normal. A period of 9 months has passed since the patient last visited the urologist, presenting with recurring symptoms including hematuria and deep bladder pain. In light of this, the medical practitioner recommended repeating sonography. In the new results of sonography, the thickness of the bladder was 6 mm (slightly higher than normal), and a polypoid lesion measuring 16 × 32 mm was observed on the posterior wall of the bladder, inclined to the right. **(**Figure 1 **)** Post‐void residual urine after emptying the bladder was equal to 42 CC. After the second sonography, the patient was referred for a computerized tomography (CT) scan and pathological sampling for a conclusive diagnosis. The initial CT scan confirmed the results of the patient's sonography **(Figure** 2 **)**. Histological findings showed malignant lymphoma with large B germinal‐type cells without metastasis to muscle tissue. After reviewing the CT scan and histological results, the patient was referred for a positron emission tomography (PET) scan. PET Scan results indicated large B‐cell lymphoma of the bladder in primary staging and confirmed the previous data. **(**Figure 3 **).**

**FIGURE 4:**
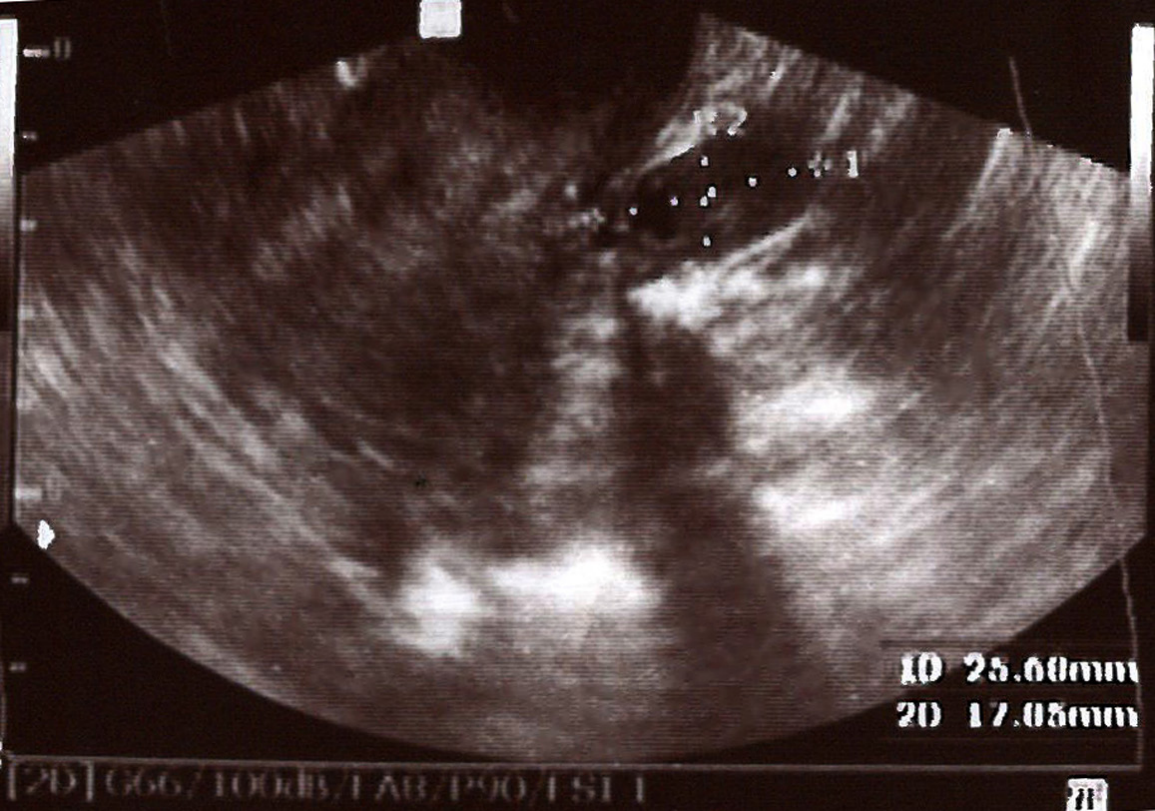
In the sonography after the radiotherapy, as can be seen, there is no trace of the lesion.

After confirming the final diagnosis, the tumor was completely removed during surgery, and the patient underwent chemotherapy for 6 months, then radiotherapy for 2 months. The chemotherapy included adrisin 50 mg, endoxan 500 mg, and vincristine 1 mg. During this period, the patient did not have a relapse of MS, although he did not receive medication to prevent it. **(**Figure 4 **).**

## 3 OUTCOME AND FOLLOW‐UP

One month after radiotherapy, the patient was referred for sonography. The ultrasound results showed that the bladder fat returned to normal (4 mm), and no manifestation of tumor recurrence was observed.

After the final ultrasound examination and the oncologist's approval, the patient's neurologist prescribed Glatiramer acetate 40 mg (brand name Cinnomer). The patient has been taking Glatiramer acetate for 4 months and has had no adverse effects, and her neurological condition is stable.

## 4 DISCUSSION

Blocking immunosurveillance of B‐cell lymphoma and myeloma via CD4 T cell is a suggestive pathophysiological pathway due to the increased risk of cancer in long‐term use of fingolimod in patients.^8^

Furthermore, based on a systematic review and meta‐analysis study that reviewed the data of 64,135 patients,^6^ the risk of cancer in pwMS using Fingolimod 0.5 mg is evaluated to be about 2.01% (95% CI: 1.00%–2.04%). This number boosted to 3.01% (95% CI: 2.02%–5.01%) in the dose of 1.25 mg, showing that increasing the drug dose can increase the probability of cancer in pwMS. Kappos et al., in 2010, shows the incidence rate of cancer with 0.5 mg of fingolimod was 0.96% compared risk of cancer with 1.25 mg were 1.39, and it shows more carcinogenic effects made by enhancing dosage. In contrast, the increasing dosage can improve the ability of medicine to suppress auto‐aggressive lymphocytes and subsequently reduce multiple sclerosis relapse probability.^2^ Also, according to a randomized controlled trial investigation that compared the risk of cancer in three medications: Fingolimod, Natalizumab, and Rituximab, unlike the other two medicines where no significant association was found between the risk of cancer and drug use, a significant relationship was observed in fingolimod.^5^

Even though previous studies have shown that Fingolimod 0.5 mg has a lower risk of cancer compared to other doses in long‐term use, in the present study, this drug was found to cause bladder lymphoma. It should be noted that according to various case reports of Fingolimod‐induced cancer, the specific point of this case is the type of cancer identified, that is, bladder lymphoma, which has not been reported by any study so far. This patient has long‐term taken of fingolimod compared to a previous study,^6^ which can increase the risk of developing cancer due to the long‐term use of the medication. Furthermore, the absence of a family history of cancer and other typical carcinogenic and mutagenic factors, such as smoking or occupational exposure to chemicals, may reduce the possibility that bladder lymphoma may be caused by factors other than long‐term Fingolimod use in this case. One of the study's limitations was the lack of biomarker tests due to imposing a high cost on the patient before and after treatment.

In conclusion, long‐term use of fingolimod has a carcinogenic effect and enhances the risk of cancer, especially, the translocation of carcinoma cells, such as in this case. However, a 0.5 mg dosage of fingolimod is safer in comparison to other dosages. Therefore, physicians should monitor their patients for cancer and replace their medicine with monoclonal antibodies for long‐term use.

## AUTHOR CONTRIBUTIONS

**Saeed Vaheb:** Conceptualization; data curation; methodology; project administration; validation; writing – original draft; writing – review and editing. **Elham Moases Ghaffary:** Methodology; project administration; visualization; writing – original draft; writing – review and editing. **Vahid Shaygannejad:** Conceptualization; data curation; investigation; methodology; project administration; supervision; validation; visualization; writing – review and editing. **Omid Mirmosayyeb:** Methodology; project administration; supervision; validation; writing – original draft; writing – review and editing.

## FUNDING INFORMATION

We do not have any financial support for this study.

## CONFLICT OF INTEREST STATEMENT

The authors have no conflict of interest to declare.

## CONSENT

Written informed consent was obtained from the patient to publish this report in accordance with the journal's patient consent policy.

## Data Availability

Data during the current study are available upon request with no restriction.
